# Scaffolding Close Reading of Mathematical Text in Pre-service Primary Teacher Education at the Tertiary Level: Design and Evaluation

**DOI:** 10.1007/s10763-022-10309-y

**Published:** 2022-09-02

**Authors:** Sebastian Rezat, Sara Malik, Markus Leifeld

**Affiliations:** Institute of Mathematics, Paderborn University, Warburger Str. 100, 33098 Paderborn, Germany

**Keywords:** Close reading, Reading strategies, Scaffolding, Teacher education, Mathematical text

## Abstract

Even in the digital age, learning mathematics at an academic level still requires much reading of mathematical text. Research has shown that reading mathematical text requires readers to engage with all the structures of the book and with its pedagogical voice, making connections, and plausible reasoning. Specific practices and strategies that support the close reading of mathematical text have been suggested; however, descriptions and empirical evaluations of materials designed to support these activities are rare. We present the design and first evaluation cycle of materials developed in a design research project that aims to scaffold close reading of mathematical text. The materials were designed and evaluated in a German university course on elementary geometry for first-year teacher education students who study mathematics to become primary teachers. The reading strategies were explained and modeled for students in reading-strategy videos. Additionally, close reading of mathematical text was scaffolded by close-reading tasks and homework tasks and problems that build on the reading strategies and were specifically designed to foster understanding of the mathematical text. Survey data were collected from 296 students to evaluate their use of and attitude toward the different materials. The quantitative results indicate that students used the materials and were generally able to learn the course content by themselves. From all provided materials, they found the close-reading tasks most helpful. A qualitative analysis of answers to open questions revealed issues with different materials, particularly with the script, and requests for additional materials. The issues with the script were categorized inductively. The categories are presented as a qualitative result of the study and discussed.

**Supplementary Information:**

The online version contains supplementary material available at 10.1007/s10763-022-10309-y.

## Introduction

Even in the digital age, the learning of mathematics at an academic level still requires much reading of mathematical text. Distance learning during the COVID-19 pandemic is likely to have increased the amount of reading in learning university-level mathematics. While the question of mathematical text requiring a specific reading ability is not exhaustively answered (Österholm, 2006), previous research has unveiled issues in which mathematical text differs from other texts (Österholm, 2006; Österholm & Bergqvist, 2013). Furthermore, differences in reading behavior among disciplines (Paul, 2018; Shanahan et al., 2011) and successful reading behavior related to mathematical text (Berger, 2019a; Shepherd et al., 2012; Shepherd & van de Sande, 2014) have been empirically identified. There are several suggestions on how reading mathematical text can be fostered by different reading strategies and teaching scenarios (Barton & Heidema, 2002; Carducci, 2020; Fenwick, 2001; Fuentes, 1998; Hubbard, 1990). Many of these suggestions are either theoretical or reports of teaching practices that the authors considered helpful, with neither having been subject to empirical evaluation based on scientific principles.

In this paper, we want to contribute to the scientific understanding of how mathematical text reading skills can be fostered at the tertiary level. We describe the design principles of how we incorporated the teaching of reading strategies in a course of elementary geometry for first-year teacher education students who study mathematics to become primary teachers. We further present the results of the first evaluation of the strategy training. We seek to answer the following questions:

1. How to scaffold students’ reading of mathematical text?
2. How do first-year teacher education students evaluate the scaffolds for reading mathematical text designed for a university course in elementary geometry?

First, we summarize the relevant literature on characteristics of mathematical reading literacy and how it can be fostered with the aim to derive design principles for our materials. Second, we describe the design methods and the methods of the evaluation of our materials. Third, we present the results, which comprise the design of our materials and the evaluation results. Finally, we discuss these results and draw some conclusions for the further development of our materials and future research on the teaching of reading strategies.

## Literature Review and Theoretical Framework

During their university studies, students are confronted with a variety of texts to be read and comprehended. Two main approaches can be distinguished to support students’ understanding from reading: a defensive and an offensive approach. A defensive approach relates to lowering the linguistic demands of the text, and an offensive approach seeks to empower students to read the text as it is by making linguistic demands a matter of learning (Leiss & Plath, 2020). While defensive approaches may be helpful to support understanding of one specific text, offensive approaches aim to empower students to read other mathematical text as well. Thus, offensive approaches contribute to students’ reading literacy and lifelong learning. Related to the offensive approach and in line with the rise of constructivist and transactional views of reading, in which “reading involves each reader actively in the transformation of the text read, mediated by his/her past experiences, knowledge and beliefs as well as the context and purpose of the reading experience itself” (Borasi & Siegel, 1990, pp. 9–10) the importance of reading strategies has increased. In our work, we define reading strategy as “the mental operations involved when readers approach a text effectively to make sense of what they read” (Barnett, 1988, p. 150). Thus, they are supposed to support the transformation process and the construction of meaning from the text.

Reading strategies are applied in three stages of reading: pre-reading, while-reading, and post-reading (Nordin et al., 2013). This requires an awareness of reading and a willingness to infer the content of the text being read. For this reason, a plan to engage with the text is developed before the actual reading (Nesbit & Adesope, 2006; Paul, 2018; Shanahan et al., 2011). While reading, the reader is aware of intrinsically understanding what is being read. The strategies used are intended to help comprehend content by asking questions and reading frequently (Carducci, 2020; Hubbard, 1990; Paul, 2018; Shepherd & van de Sande, 2014). However, the comprehension of the text does not end with the actual reading. For a deeper understanding, readers should summarize important information of the text after reading, thereby enabling reflective inferences about the content (Nordin et al., 2013).

### Subject- and Genre-Specific Reading Strategies

Widely known general reading techniques and methods integrate different reading strategies related to the three stages of reading into one master method. For example, the PQ4R method (Thomas & Robinson, 1972) denotes a complex reading method consisting of different stages and related strategies (**p**review, **q**uestion, **r**eading, **r**eflect, **r**ecite, **r**eview) that can be divided into strategies related to the three stages: pre-reading (preview, question), while-reading (reading), and post-reading (reflect, recite, review). Another example is the MURDER technique (Dansereau et al., 1979) that consists of different stages and related strategies (**m**ood, **u**nderstand, **r**ecall, **d**etect, **e**xpand, **r**eview) that can also be divided into the three stages: pre-reading (mood), while-reading (understand, recall, detect), and post-reading (expand, review). While these methods are supposed to apply to every text, researchers have become more aware of the specific disciplinary characteristics of text (Rezat & Rezat, 2017) and related reading requirements. Therefore, differences in reading literacy in different disciplines have been investigated (Österholm, 2006; Paul, 2018; Shanahan et al., 2011). Österholm (2006) concluded that.

there is no common type of reading comprehension for mathematical texts in general, but one seems to need several types of skills for different types of mathematical texts. In particular, mathematical texts using symbols seem to demand a special type of skill for reading comprehension. In contrast, mathematical texts written in natural language do not seem to need any special type of skill except a more general reading ability. (pp. 340–341)

Besides differences in reading literacy, subject- and genre-specific reading strategies have been empirically investigated such as the reading of (university) mathematics textbooks (Berger, 2019a, b; Österholm, 2008; Shepherd et al., 2012) or the reading of mathematical proofs (Panse et al., 2018; Weber, 2015). From these studies, it is apparent that successful reading of mathematical text is achieved by what has been termed *close reading*. Berger (2019a) described a close reader of mathematical text as a reader who applies the following strategies: *skimming* to see what is known and what is new; *careful reading of the entire text*, including definitions, theorems, proofs, worked examples, and highlighting what is new; *making connections* by relating what is read to relevant theorems, worked examples, or to prior knowledge; and *working examples and exercises* by doing worked examples on one’s own and comparing with the solution provided in the textbook or solving exercises after reading the text. These strategies may be allocated to the different stages of reading distinguished by Nordin et al. (2013). Skimming is a pre-reading strategy, careful reading of the entire text and making connections is what is done while reading, and examples and exercises are solved post-reading.

To deepen the understanding of what characterizes close reading, studies investigating the behavior of expert readers in mathematics provide further insights. Shepherd and van de Sande (2014) examined differences between novice and expert readers in mathematics. They found differences in (a) mathematical fluency, (b) comprehension monitoring strategies, and (c) how readers engaged with the exposition. In particular, more mathematically advanced readers showed higher mathematical fluency, which includes.

a familiarity with technical terms and symbols, the ability to skim over information that they found easy to assimilate, and the ability to instantaneously translate symbol configurations into meaning [; … furthermore, they] checked their comprehension more frequently and more deeply, were willing to spend time constructing understanding voluntarily, and were ready to persevere when confronted with material that they found confusing or difficult to assimilate. (Shepherd & van de Sande, 2014, p. 84)

Finally, more mathematically advanced readers explored the content using multiple representations (e.g. figures, tables, graphs) and through their creation of examples. In their study of differences in disciplinary reading processes of expert readers in three disciplines (i.e. history, chemistry, mathematics), Shanahan et al. (2011) found that expert readers in mathematics reconcile the text with their knowledge to focus attention, use text structure to support understanding, and make no distinction between graphical elements and prose; however, they conceive text in a unified manner, put a strong emphasis on accuracy, and reread rigorously and intensively to weigh all information.

In summary, close reading is characterized by a set of strategies that successful readers apply to understand mathematical text. It comprises skimming for new and challenging to understand information in the pre-reading stage; precise and careful reading of the whole text; making connections among previous knowledge and what is read, among different representations, and between the read contents and exercises to construct understanding while reading; and working examples and exercises in the post-reading stage.

Of all mathematical genres, the reading of proof has received particular attention. In an exploratory study, Weber (2015) identified six strategies to read proof elicited by mathematically advanced and experienced students: “Understand the Theorem Statement” (p. 296), “Try to Prove the Theorem Statement Before Reading its Proof” (p. 297), “Considering the Proof Framework Used in the Proof” (p. 298), “Partitioning the Proof into Parts or sub-Proofs” (p. 299), “Using Examples to Make Sense of Statements Within the Proof” (p. 301), and “Comparing the Method in the Proof to one’s own Methods” (p. 302). Panse et al. (2018) investigated if reading proof for validation or for comprehension results in different reading behavior; they found that the reading behavior of mathematical proof was not affected by the two different goals. However, they concluded that “instructors might well wish to promote effective mathematical reading … and to use specific tasks both to teach reading skills and to test the success of their efforts” (Panse et al., 2018, p. 371). The question remains: What could these tasks look like or, more generally, how can close reading of mathematical text be scaffolded or learned?

### Teaching the Reading of Mathematical Text

Comparatively little research has been conducted to investigate how the learning of reading strategies can be effectively implemented and incorporated into the learning of mathematics. Hubbard (1990) distinguished three methods of improving reading skills:

1. Students can be provided with a handout on mathematical reading and study skills, which they must read for themselves. 2. Students can be offered a self-contained reading and study skills course at the beginning of their tertiary studies. 3. Reading and study skills can be incorporated into normal coursework by the regular lecturer on an ongoing basis (p. 266).

In an intervention study, Hagena et al. (2017) designed a 15-week reading strategy training to foster students’ competency in mathematical modeling. They investigated three different conditions that related to the differentiation by Hubbard (1990): integrated reading-strategy training, separate reading-strategy training, and no strategy training. Their study did not yield any results in favor of one of the three conditions.

Fisher and Frey (2016) suggested an instructional approach for learning how to read complex texts, which they termed “close reading” (p. 403) and that comprises six instructional practices: “(1) learning intentions, (2) teacher modeling, (3) close reading, (4) scaffolded reading, (5) text-based collaborative conversations, and (6) wide reading” (p. 404). From these, teacher modeling is of particular interest because it adds another practice to the methods of improving reading skills summarized by Hubbard (1990). This additional practice aims at teaching reading strategies by allowing students to witness how an expert proceeds when reading a text for understanding by thinking aloud while reading.

In summary, we find two strands of research in the relevant literature. On the one hand, there are empirical investigations of the strategies applied by expert readers; on the other hand, we find suggestions of methods that foster the reading of mathematical text. However, the first strand does not suggest how to learn these strategies; in the second strand, there is no empirical evidence that supports the advantage of one method over the others. Empirical evaluations of reading strategy training are rare. Therefore, in our approach, we aimed to connect these two strands of research by integrating Fisher and Frey’s (2016) suggestions of a method for teaching close reading with the empirical insights by Berger (2019a, b) into the strategies of close reading by expert readers. Our goal was to integrate teaching experiences and empirical insights in a productive way.

## Methodology

Our study is methodologically grounded in design research (Gravemeijer & Cobb, 2006). In this section, we provide details about the participants, describe the design of the materials and related principles, and present the instruments used in the evaluation of the materials.

### Participants

Students in the first year of their teacher education program for the primary level at a German university were invited to participate in our study; in response, 296 students completed the questionnaire. The students had finished the upper secondary level after at least 12 years of schooling and, therefore, are qualified to enter university. Considering the German KMK (https://www.kmk.org/) educational standards, it can be expected that students have the following prior knowledge in geometry that is particularly relevant for this teaching concept: experience in describing and justifying properties and relationships of geometric objects (e.g. symmetry, congruence, similarity, positional relationships). They can use this knowledge in the context of problem-solving to analyze relationships. It must be considered that this knowledge was acquired mainly at the lower secondary level because upper secondary level lessons focus on analytical geometry or linear algebra.

### Design of Materials and Related Principles

Our overall intention in the design of the materials was to scaffold students’ practices of close reading of mathematical text, as described by Berger (2019a), with the goal of fostering their comprehension of mathematical text (i.e. the construction of a mental representation of the mathematical content; Österholm, 2006). To achieve this, we adopted instructional practices suggested by Fisher and Frey (2016) to foster close reading. We developed a set of materials with different aims to implement these instructional practices in different stages of reading. For each weekly portion of the script, we focused on one close-reading strategy from the literature that we thought could be particularly helpful for the comprehension of the script content. This strategy was then exemplified in the materials closely linked to the content.

### Questionnaire

After implementing our materials, we surveyed the students’ use of and beliefs about them. For this purpose, we developed a questionnaire that was used halfway through the semester. The questionnaire consisted of 24 Likert scale and two open questions. The Likert scale comprised six levels, and the following meanings were assigned: 0 = does not apply at all and 5 = applies completely. In this section, we do not present all the questions in detail but summarize the focal points of those questions that we consider relevant. Students were asked to what extent they understood the organization of the course. In other questions, they were asked to assess the extent to which they could work through the course content by themselves or, if necessary, with recourse to the materials provided. The main part of the questionnaire asked about the different course materials. Students were invited to evaluate the comprehensibility and degree of difficulty as well as how they used the materials. The questionnaire contained an open question to provide students the opportunity to give feedback on issues that occurred when working with the different materials.

### Data Analysis

We analyzed the Likert-scaled items by methods of descriptive statistics. The 198 answers to the open questions were analyzed based on qualitative content analysis according to Mayring (2015). Categories were developed inductively from the data by applying the procedures described by Mayring. The category system was developed in a two-stage process. In the first step, the material was reviewed according to the issues with the different materials and requests for additional scaffolds that students mentioned in their answers. In the next step, the issues and the requested scaffolds were divided into more differentiated descriptive categories. The second author mainly developed the categories and discussed these with the other authors. After developing the category system, the data were coded and analyzed quantitatively based on the categories. To ensure reliability, student answers were coded by the second and the third authors independently. We used Cohen’s kappa to determine interrater reliability as we had two raters and mutually exclusive categories. For categorizing how answers related to the different materials, issues with the script, and student requests for further supporting materials, Cohen’s kappa values were κ_1_ = 0.97, κ_2_ = 0.93, and κ_3_ = 0.91, respectively. These values can be considered an almost perfect agreement.

## Results

### Designing for Scaffolding Close Reading of Mathematical Text

In this section, we answer research question 1 by describing the design of our materials. The materials addressed first-year university students studying mathematics to become primary teachers. The course content was elementary geometry. Appendix A provides an overview of the course content and related reading-strategy videos.

**Fig. 1:**
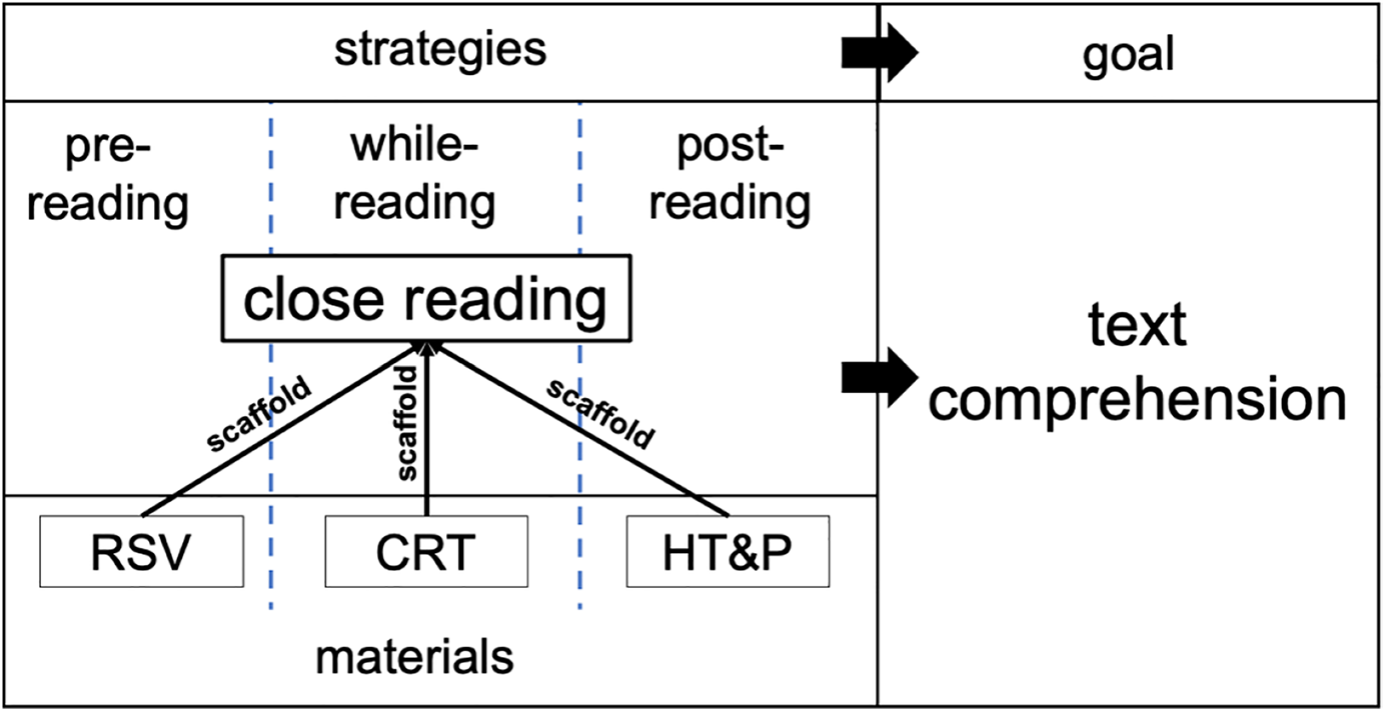
Overall structure of the scaffolding concept of the course and related materials. *RSV* reading-strategy videos, *CRT* close reading tasks. *HT&P* homework tasks and problems

On the level of the overall course design, we developed a set of materials with different aims. Our materials comprised a script of the course content, reading-strategy videos (RSV), close-reading tasks (CRT), and homework tasks and problems (HT&P). Each of the materials aimed at implementing different practices of close reading in the different stages of reading; therefore, it was necessary to develop different materials to be used in each stage. Those materials are described below. Figure 1 provides an overview of the overall structure of the materials and shows in which stage they were designed to scaffold close reading.

#### Script

The script was the primary material to communicate the content of the course. The script was initially written by a colleague, Peter Bender of Paderborn University, and revised by the authors to focus on language issues. It was provided to the students in weekly portions of 3–8 pages. A two-page sample of the script for week 3, “Composition of isometries,” is provided in Appendix B. We selected this generic example because it offers a good insight into the materials’ typical characteristics and how they related to the specific script content.

As previous research has shown that reading mathematical symbolism requires special reading abilities (Österholm, 2006) and students often have difficulty with formal mathematical symbolism (Österholm, 2008), we considered that this would be especially relevant for first-year students who must become familiar with formal mathematical writing. Therefore, we used a defensive approach, mentioned earlier, and sought to reduce the symbolism to relate to students’ general reading ability; we expressed the concepts, rules, and procedures as much as possible in natural language. Since it is neither possible nor reasonable to eliminate all symbolism from the text, it still contains some symbolism to introduce students to its reading. We inserted links to the related materials (e.g. RSV, CRT, HT&P) in the text.

#### Reading-Strategy Videos (RSVs)

The main aim of the RSVs is to demonstrate how an expert reader would read a given portion of the script in the pre-reading phase. Research has shown that reading mathematical text requires different practices and strategies than other texts. Supposedly, students in their first year at university are unfamiliar with these practices and strategies. Therefore, we decided that model-learning (Bandura, 1986) provides a good background theory to introduce these practices and strategies to students. Over the course, we produced several RSVs (see Appendix A for an overview). Adopting the close-reading approach by Fisher and Frey (2016), the RSVs aimed to scaffold students’ close reading of the script by modeling an expert reader’s strategies. Each video focused on one specific reading strategy that we thought could be exemplified in a specific excerpt of the script and would help scaffold its close reading. At the same time, the reading strategies were intended to be generic and applicable to other portions of the script. Here, we describe one paradigmatic example—design of the RSV “understanding mathematical argumentation”—in more detail; its relevance was described in the literature review.

The week 5 script excerpt contained eight conclusions drawn from the theorem about the composition of two reflections. Each conclusion is justified by a short proof or some kind of mathematical argumentation. Therefore, we chose “understanding mathematical argumentation” as a central theme when reading this excerpt. The RSV aimed at modeling how an expert would read mathematical proofs and argumentations. Therefore, we drew on the strategies identified by Weber (2015). However, due to our students’ lack of mathematical experience, we decided that it would be too difficult for them to model every strategy of close reading of mathematical proof in our RSV. In particular, we thought that strategies that required students to prove the theorems on their own or consider the proof framework were not appropriate for our students. Instead, we focused on more basic strategies such as “understand the theorem, … partitioning the proof into parts, … [and] using examples to make sense of statements within the proof” (Weber, 2015, pp. 296–301). We then combined these with a demonstration of selected aspects of close reading of mathematical text as described by Berger (2019a), Shanahan et al. (2011), and Shepherd and van de Sande (2014).

The RSV starts with a short introduction to the if–then structure of mathematical statements. To support understanding of mathematical statements in different mathematical texts, we introduced alternative wordings and structures of mathematical statements (e.g. statements with reversed order [not logically] of premises and conclusion in the phrasing). This information about the structure of mathematical theorems is exemplified in the script by highlighting the premises in green and the conclusion in red. We aimed to provide students with the relevant background knowledge to understand the theorem statement (Weber, 2015). Then the speaker in the video demonstrates how to read the proof of a mathematical theorem. The speaker stops after each sentence and explains how he makes sense of the sentence by relating symbols and statements about relations to the geometrical figure. This was supposed to demonstrate how to spend time on constructing understanding (Shepherd & van de Sande, 2014) by conceiving text in a unified manner (Shanahan et al., 2011) and making connections (Berger, 2019a) as an aspect of close reading. The speaker further exemplifies certain statements by carrying out related constructions in GeoGebra and, thus, demonstrates how to generate examples as another critical aspect of close reading (Shepherd & van de Sande, 2014; Weber, 2015). Finally, the speaker exemplifies how he stops after each statement to think of a justification for this statement. This demonstrates how to partition the proof into parts (Weber, 2015) and check comprehension frequently and deeply (Shepherd & van de Sande, 2014). An overview of the structure of the RSV with related modeling of strategies for close reading is provided in Appendix C.

#### Close-Reading Tasks (CRTs)

The aim of the CRTs is to scaffold close reading by providing tasks that should be solved while reading. Berger (2019a) suggested that making connections is an essential close-reading strategy, while Fisher and Frey (2016) proposed that reading needs to be scaffolded. To engage the reader actively in making connections, we designed the CRTs closely linked with the mathematical content. While the RSV aimed at modeling the practice of a close reader for more extensive text passages, we inserted a CRT wherever we thought it was applicable. They were supposed to interrupt the flow of reading and engage the reader in activities that close readers would typically do at that point to check their comprehension and to spend time constructing understanding (Shepherd & van de Sande, 2014). The CRTs comprise creating own examples (Shepherd & van de Sande, 2014), applying a rule, or carrying out mathematical problem-solving strategies that were developed and described earlier. The CRT aimed at implementing what Panse et al. (2018) suggested for fostering the reading of proof.

**Fig. 2:**
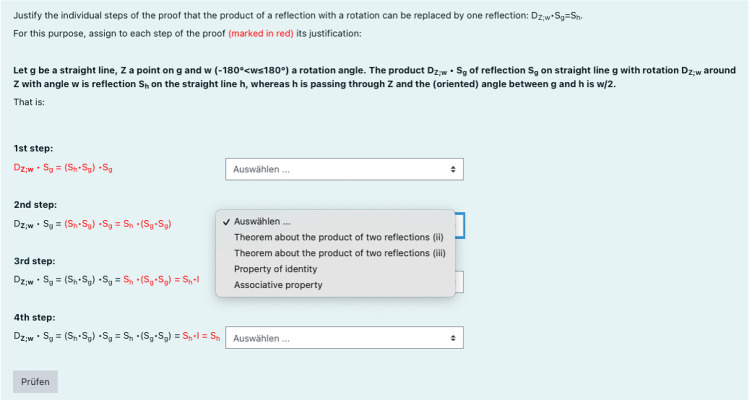
Example of a close-reading task

Here, we describe CRT no. 7 (Fig. 2) in more detail. It is related to the script excerpt and the RSV described in the previous sections. The CRT starts with the statement of the task, followed by the theorem taken from the script to be proved. The CRT relates to the conclusion (v) and its proof in the script. Conclusion (v) says: Let *g* be a straight line, *Z* a point on *g*, and *w* $(-180^\circ <w\le 180^\circ )$ a rotation angle. The product ${D}_{Z;w}\circ {S}_{g}$ of reflection ${S}_{g}$ on straight line *g* with rotation ${D}_{Z;w}$ around *Z* with angle *w* is reflection ${S}_{h}$ on the straight line *h*, whereas *h* is passing through *Z* and the (oriented) angle between *g* and *h* is $\frac{w}{2}$. The proof comprises both cases: ${D}_{Z;w}\circ {S}_{g}$ and ${S}_{g}{\circ D}_{Z;w}$. The main idea is that ${D}_{Z;w}$ is substituted by two reflections passing through *Z* with angle $\frac{w}{2}$ between *g* and *h*. How the resulting product of three reflections is reduced to the reflection ${S}_{h}$ is proved in a chain of equations. The chain of equations is partitioned into four steps. Each step requires students to choose a justification for the equality from a drop-down menu. They are required to stop at each equal sign of this chain of equations and apply the justification strategy as demonstrated in the RSV by answering why the equality is true. Therefore, students cannot simply read over the chain of equations but must check their comprehension by thinking more deeply about the validity of each step. The CRT provides automated feedback about the correctness of their answers. This CRT scaffolds students in applying the strategy to justify every equal sign in the proof that was modeled in the RSV (Appendix C: 12:17–16:02) .

#### Homework Tasks and Problems (HT&Ps)

**Fig. 3:**
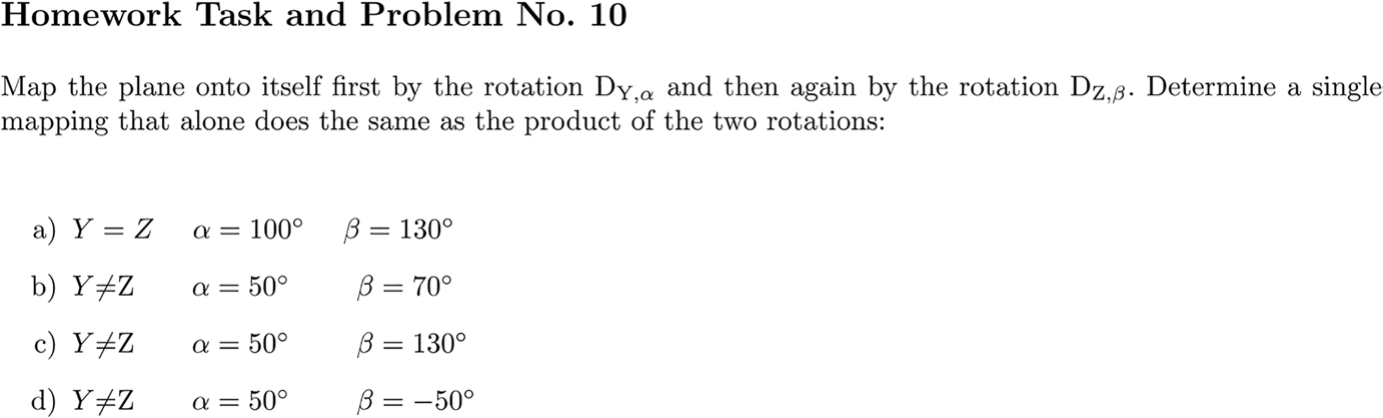
Homework task and problem no. 10 related to the script excerpt in Appendix B. **Notice: **Except for a), take a source triangle (figure A), construct its target figure (figure B) at $D_{Y,\alpha}$. Take figure B as the new source figure and construct its target figure (figure C) at $D_{Z,\;\beta}$ . (One can start the constructions for b), c) and d) in the same way: the same point Y, the same point Z, the same triangle and the same first rotation each time; only for the second rotation $D_{Z,\;\beta}$ it works differently for b), c) and d)). Next, we look for the mapping that directly assigns the source points to the final target points. In order to find it, you first have to determine the angle between the line of the final target triangle (figure C) and the corresponding lines of the source triangle (figure A)

The HT&Ps were designed to be solved by groups of three students and to foster text-based collaborative conversations in the post-reading phase. The aim of the HT&Ps was to combine the practice of promoting text-based collaborative conversations suggested by Fisher and Frey (2016) while working examples and exercises, which Berger (2019a) had considered an essential practice of close reading. The scope of the HT&P was broader than that of CRTs as the latter focused only on small passages of the script excerpt. Students were supposed to transfer the reduction strategy that was presented and proved in the script and used as an example in the RSV to four different examples that cover different cases. Figure 3 presents an example of a HT&P related to the script excerpt presented in Appendix B.

### Students’ Evaluation of the Materials

In this section, to answer research question 2, we present the results of the students’ evaluation of the materials. The results are related not only to the content described earlier but also to the content of the first half of the course. We present the quantitative results of our survey; also, the students’ answers to the open questions of the evaluation (i.e. What problems do you have when working with the individual materials [script, reading-strategy videos, close-reading tasks, homework tasks and problems]?) are systematized and discussed based on qualitative content analysis.

#### Quantitative Results

Table 1 presents the results of how students evaluated the handling of the materials and the whole process of learning the course content through reading. The percent values in the six columns represent the proportion of students who agreed with the statement in the respective degrees from 0 (do not agree at all) to 5 (agree completely).

**Table 1:** Results from the survey questionnaire

Question	0 (%)^*^	1 (%)	2 (%)	3 (%)	4 (%)	5 (%)	*n*	*M*	*SD*
I can manage the digital lecture materials as a complete package (script, reading-strategy videos, close-reading tasks, homework tasks and problems)	3	8	13	30	32	13	294	3.20	1.10
I am able to understand the content of the course by using the different materials (script, reading-strategy videos, close-reading tasks, homework tasks and problems)	3	6	17	33	34	8	295	3.09	1.15
I feel over-challenged with learning the course content by myself	5	19	20	23	23	10	294	2.68	1.40
I feel over-challenged with the content of the course	6	18	26	28	17	5	294	2.46	1.29
The level of the homework tasks and problems is appropriate	2	2	8	19	44	24	292	3.76	0.95
The level of the close-reading tasks is appropriate	1	5	11	29	34	20	294	3.49	1.07
The close-reading tasks support me in comprehending the content of the script	3	4	9	24	30	30	294	3.62	1.11
I apply the reading strategies presented in the videos to access the script	2	3	13	18	30	33	294	3.69	1.15
The reading-strategy videos help me to understand the content of the script	5	11	24	28	20	12	291	2.82	1.18

^*^0 = do not agree at all – 5 = agree completely

We combined levels 0 and 1 of the Likert scale as expressions of disapproval and levels 4 and 5 as affirmative answers to the given statements. Results indicated that 45% of the students were able to manage working with the different digital lecture materials as a complete package while 42% confirmed that they were able to understand the course content by using the different materials; this shows that the overall design of the course and its content were manageable for almost half of the students. However, one third (33%) of the students indicated that they felt over-challenged with learning the course content by themselves; the mean value shows that the students tended to refuse this statement on average, and the standard deviation represents a considerable variation in the data. When asked if they felt over-challenged with the course content, only 22% of the students responded affirmatively. Considering the mean value, it can be concluded that the students tended to deny this statement on average; the standard deviation also reflects a relatively large variation in the data. Thus, working through the content by themselves was a greater challenge for the students than the course content itself.

If we take a closer look at the evaluation of the different course materials, we see that the CRTs seemed to be adequate and helpful for the students; 60% confirmed that they found the CRTs helpful for developing an understanding of the script content and more than half evaluated the level of the CRTs as appropriate.

The picture related to the RSVs is somewhat different. While 63% of the students declared that they applied the reading strategies modeled in the RSVs to access the course content, only 32% confirmed that the RSVs supported understanding the script content. The mean value and standard deviation also demonstrate that, on average, the RSV supported the students less in understanding and learning the content. This result is in line with the intention of the RSV as it was not designed to explain the content itself but was aimed at modeling how students may achieve an understanding of the content by applying the reading strategies. This may indicate that students were not able to apply the reading strategies successfully to comprehend the script content.

The level of the HT&P was predominantly evaluated as appropriate. The extent to which the HT&P contributed to the understanding of the script was not evaluated in this survey because the completion and submission of weekly exercises is a fixed component of the course work and, therefore, not an innovation in our course concept.

#### Qualitative Results

In this section, we present the results of the qualitative analysis of the students’ answers to the open question: What problems do you have when working with the individual materials (script, reading-strategy videos, close-reading tasks, homework tasks and problems)? Table 2 shows how many of the students’ answers related to the respective course materials. Most of the students’ answers (70%) related to difficulty or issues with the script. Because students indicated they would appreciate additional support or scaffolds, we will address issues with the script as well as scaffolds. This is followed by a description of the problem areas that were identified in connection with the other materials; there, the quantitative distribution is not considered because the number of answers is too small for a viable interpretation. Nevertheless, the categories themselves provide insight into potential difficulties students had in using the materials.

**Table 2:** Numbers of questionnaire answers related to the different course materials

Individual materials	*N* = 198	%
Script	139	70
Close-reading tasks	37	19
Reading-strategy videos	16	8
Homework tasks and problems	45	23
No coding	24	12

In some cases, statements were assigned to more than one category as several materials were addressed

Table 3 shows the results of the qualitative analysis of the students’ issues with the script; column 1 shows the inductively formed categories, and column 2 provides a corresponding prototypical answer exemplifying each category. Note that 36% of the students found the language of the script very demanding, complicated, and difficult to understand. A further 17% indicated that the presentation of the mathematical content was unfamiliar and very theoretical; another 14% stated that it was generally incomprehensible.

**Table 3:** Categories of issues with the script developed from the questionnaire answers to the open question

Issue	Prototypical answer	*N* = 139	%
Formulations of the script are very demanding, complicated, difficult to comprehend	The script is often written in a complicated way. The definitions in the boxes are often explained 2 or 3 times in more detail, which makes it more confusing for me	50	36
Unfamiliar presentation of mathematical content (formal, theoretical)	The script is formulated very mathematically	24	17
Assessed as incomprehensible without giving reasons	Script quite incomprehensible	19	14
Missing relationship between text and tasks	Some of the tasks do not always fit together or do not relate directly to the script	15	11
Missing examples	The script is often too theoretical for me, I miss examples. In order to understand the script, I would need a worked example, in order to then be able to work on a task by myself	8	6
Lack of visual representations	Due to the lack of visualizations, it is challenging to imagine the content presented	7	5
Questions about the script cannot be clarified	In the case of comprehension problems, there is no possibility to ask questions	5	4
Strategy cannot always be applied	The attempt to visualize the content of the script with a drawing is not always successful	1	1
Not enough videos	Not enough videos	1	1

Although we adopted a defensive approach in the development of the script, the results show that the verbalization of the content and the textual design of the script were a major challenge for these students. For example, students communicated in this context:

The script is sometimes unnecessarily complicated. Sometimes I think to myself ‘Please what?’- but then I work on the tasks on Panda [CRT provided in Moodle, which is named Panda at Paderborn University], where the content is explained in a much easier way and then I finally understand it. So, the script confuses me from time to time.

For me it is difficult to relate the read content to the exercises, because in most cases I do not understand the theoretical knowledge in the script.

Altogether 11% of the students that related to the script in their answers indicated that they would appreciate more examples and visualizations to better understand the script content. Furthermore, a similar ratio of students (11%) expressed that they did not always see the link between the task and the content.

Table 4 provides an overview of scaffolds that students believed would help them better understand the course content. Among these, explanatory videos or some kind of lecture videos were the most requested additional scaffold (19%); this indicated to us that these students would prefer a different representation of the course content than a mere textual representation. It is striking in this context that only two students (1%) expressed that they would appreciate more RSVs to better understand the text content. A few other students articulated that they would appreciate more examples (3%) and more visualizations (5%).

**Table 4:** Student questionnaire requests for additional scaffolds to foster their understanding from reading the script

Additional scaffolds	Prototypical answer	*N* = 139	%
Explanatory videos/online lecture	I would find it easier if there were more explanatory videos or exercises	27	19
Further visualization	A few more visualizations in the script would help me to understand the methods better	7	5
Further examples	I think it would be easier to understand the script if there were illustrative examples, because it is sometimes difficult for me to imagine some things correctly	4	3
More tasks	The script makes it difficult for me to understand the content. I would find it easier if there were more explanatory videos or tasks	3	2
RSV	I would like to see more reading strategy videos	2	1
Other “language”/colloquial language to explain the content	It would be very helpful to me if future videos were uploaded on the content of the lecture or the script in which the subject matter was presented in more detail, in colloquial language and, if necessary, with visualizations (e.g. drawings)	2	1
Other approach than the formal-mathematical approach	Someone who explains the script again in a different way would be helpful in many cases	1	1
Possibility to ask questions	A general opportunity to discuss the script and open questions (preferably during the scheduled lecture time) would help me a lot here	1	1
Glossary	It would be nice to have more individual definitions of terms/glossary	1	1
Further explanations of the content	I think that the script could be expanded in some places	1	1

In some cases, statements were assigned more than one category because several categories were addressed in the answers

Regarding the other materials, 16 students expressed no problems with the RSVs. For the CRTs and the HT&Ps, students stated that it was sometimes difficult to establish references to other tasks already dealt with or to text passages in the script. For both materials, they expressed that the tasks were sometimes unclear and difficult to work on.

## Discussion and Conclusion

In this paper, we describe the design of a university course in elementary geometry for first-year primary teacher education students that aimed at scaffolding students’ close reading of mathematical text. As an answer to our first research question *How to scaffold students’ reading of mathematical text?*, we described the set of materials and related design principles for scaffolding students’ close reading. Our materials aimed to scaffold close reading in different stages of the reading process (i.e. pre-, while-, and post-reading). We developed a script with less use of mathematical formalism and symbolism to present the course content, reading-strategy videos for the pre-reading phase that model reading strategies of expert mathematical readers, close-reading tasks to activate students in making connections while reading mathematical text, and homework tasks and problems to support understanding of the course content in the post-reading stage. These materials are interrelated and closely linked with the content of a particular portion of the script. Nevertheless, the materials are generic in the way that the main idea, structure, and organization could be adapted to other mathematics content or courses. Furthermore, most of the close-reading strategies developed in the RSV could be exemplified with other mathematical content.

Our design principles build on instructional methods for the teaching of close reading and on reading strategies observed with expert readers of mathematical text from previous research. Combining a defensive approach for the language demands of the script with a set of materials that aim to implement different reading strategies at different stages of reading can be seen as a novelty of our approach. Especially, modeling the reading behavior of an expert reader in the RSV and activating students to apply this behavior in the CRT is an innovative course design for fostering mathematical reading literacy.

We put particular emphasis on describing the design of our materials as we see this as a first and important step to contributing to a scholarly foundation of educational design. The lack of scholarship in educational design has been identified as a dearth of research by several authors (e.g. Schunn, 2008). This way, other research can systematically relate to our design decisions and build on them.

As an answer to our second research question *How first-year teacher education students evaluate the materials?*, our quantitative and qualitative evaluation of students’ use of and beliefs about these materials show once more that learning mathematics by reading is very challenging for students. Nevertheless, almost half of the students felt able to acquire the course content using the materials. Our evaluation indicated that the RSVs and CRTs were considered good means by almost half of the students to support understanding, yet they are not sufficient so far.

Although we used a defensive approach and reduced formal mathematical language and symbolism, many students found the script to be problematic. The language used in the script was evaluated as demanding, complicated, difficult to understand, formal, and unfamiliar. The students expressed that they would appreciate if the script content was presented and explained differently in a lecture or explanatory videos. Our interpretation of this request is that students would prefer materials that provide other ways to access the script content by requiring less effort in reading and understanding from reading. However, our course had two main aims: students learning elementary geometry and fostering students’ mathematical reading skills (i.e. their ability to make sense of mathematical text through reading). Therefore, exclusive access to the content through reading was pivotal for the design of the course. Extending the defensive approach by further lowering the demand of the mathematical text also seems inadequate as the goal is to empower students to be able to read mathematical text of a given complexity. Therefore, adapting the text to a level that students can understand without difficulty is not conducive in this regard. Nevertheless, the student evaluations show that they encountered obstacles when trying to make sense of mathematical text; therefore, we must put further efforts into making the presentation of the script content more accessible through reading. The students hinted how this could be achieved: one tenth asked for more examples and visualizations. Elaborating on the script content by referring to further examples and providing more visualizations could be the first step to improving the script by keeping the demand on the same level. Nevertheless, we conclude that we need to develop further insight into students’ particular difficulties with mathematical text to improve the textual representation of mathematics and provide scaffolds that are better tailored for students’ needs.

In the survey, students indicated that they would appreciate more opportunities to discuss the script content and ask questions. While our HT&Ps were meant to provide peer collaboration and peer discussion opportunities, there seems to be an additional need in this area. Providing room for questions and discussion with an expert might be a different format that allows students to talk about the script content in their own words and open space for less formal language.

One important finding from our study is that students considered the CRTs particularly helpful. Therefore, we regard this type of task that is very closely related to short passages of the text as an essential design element that needs to be preserved and further elaborated.

Many students used the RSV, but the quantitative results suggest that it was challenging for students to apply the reading strategies to increase their understanding through reading. Different interpretations of this result are possible. On the one hand, this could mean that the quality of our RSV was not good enough to be helpful for students; in this case, the next step would be to improve the quality of our RSV. On the other hand, this result could indicate that merely modeling reading strategies in videos is not sufficient. Students might need further assistance in the application of the reading strategies. Consequently, we would need to think of possibilities to practice the reading strategies modeled in the RSV. Either way, more in-depth studies are necessary to understand better the obstacles to applying the RSV reading strategies. This implies that we need to develop a more differentiated view of how students work with each RSV and how they can apply the different reading strategies. It could be that some reading strategies are better to apply than others and that the obstacles to using the reading strategies vary according to the strategy.

In summary, the results indicate that materials in our course design were evaluated as beneficial and that possible amendments be made to our set of materials. Furthermore, students’ evaluations proved once more that the language and presentation of mathematics confront students with difficulties. In particular, there is a need for further research to understand better the difficulties and obstacles that students encounter when reading the script and applying the reading strategies from the RSVs. In the next step, we aim to get a more detailed account of what students indicated as incomprehensible, demanding, complicated, or difficult to comprehend by asking them to highlight and comment on difficult-to-understand script passages. These data will be analyzed and used to prepare a lecture. The lecturer will elaborate more extensively on the passages that a critical number of students marked as difficult to understand. This lecture will also allow students the opportunity to ask questions and discuss the content. We plan to investigate how students work with the RSV in detail by observing and interviewing them when watching and applying the modeled reading strategies.

## Supplementary Information

Below is the link to the electronic supplementary material.

Supplementary file1 (PDF 716 KB)
