# Supplementary material for: Scaffolding Close Reading of Mathematical Text in Pre-service Primary Teacher Education at the Tertiary Level: Design and Evaluation
Source: Int J Sci Math Educ. 2022 Sep 2;20(Suppl 1):215–36. doi: 10.1007/s10763-022-10309-y (PMC9438878; doi:10.1007/s10763-022-10309-y)
Supplement: Supplementary file 1 — Supplementary file1 (PDF 716 KB) [file 10763_2022_10309_MOESM1_ESM.pdf]

Appendix A

Overview of the Contents of the Course and Related Reading-strategy Videos

Title	Content	Reading-strategy video
1. Congruence and isometries of the Euclidian plane	Definition of congruence. Definition of the isometries of the Euclidian plane.	Understanding mathematical concepts by examples and counterexamples
2. Isometries and symmetry	Characteristics of isometries. Invariance and fixpoints of isometries. Definitions of symmetry and symmetry group.	Understanding mathematical concepts and rules by case-by-case analysis
3. Compositions of isometries*	Compositions of isometries. Compositions of two reflections (including proof). Three-reflections-theorem (including proof).	Understanding mathematical statements and argumentation while-reading
4. Frieze groups	Definition of frieze patterns. Seven frieze groups.	
5. Platonic tessellations	Definition of (Platonic) tessellation. Number of Platonic tessellations (including proof).	
6. Archimedean tessellations	Definition of Archimedean tessellation. Number of Archimedean tessellations (including proof).	
7. (Platonic and) Archimedean solids	Definition of Platonic and Archimedean solids. Characteristics of Archimedean soli's: Euler's polyhedral formula.	
8. Existence and completeness of (Platonic and) Archimedean solids	Proofing the existence and total number of Archimedean solids. Construction of Archimedean solids from Platonic solids.	Visualization I (based on using provided materials, e.g., polygons are visualizations in the text)
9. Dual-Archimedean solids	Definition of dual-Archimedean solids.	Visualization II (based on building 3d-models or projections of 3d-models in GeoGebra)
10. Symmetry of (dual-) Archimedean solids	Symmetry of Archimedean solids and their dedicated dual-Archimedean solids.	Visualization III (based on operating with given 3d-models)

* focus in this paper

Appendix B

Sample of the Script

Theorem: The **product of two reflections** Sg (first mapping) and S_h (second mapping) is:

(i) the translation $V_{2\cdot a}$ (with length 2·a) (with the identity as a special case, if g = h), if g and h are parallel and have the (oriented) perpendicular a (vector with length a≥0) from g to h.

(ii) the rotation $D_{Z;\, 2\cdot w}$ (with the "point reflection" as a special case for w = 90°), if g and h are not parallel, but have a well-defined intersection point Z and enclose the (oriented) angle with measure w ≠ 0 ° (between 90° exclusive and 90° inclusive) from g to h.

(iii) the identity, if g = h (special case of (i) with a as the zero vector, i.e. a = 0; can also be treated like (ii) with g = h, i.e. w = 0 ° and some center Z).

(iv) If you reverse the order of the first and the second mapping, then $V_{-2\cdot a}$ or $D_{Z;\, -2\cdot w.}$

Understanding mathematical statements and argumentations while-reading.

Conclusions:

(i) In general, a composition of two reflections is not commutative. In only two cases $S_h \bullet S_g = S_g \bullet S_h$: If g=h it is the identity, and if g and h are orthogonal, then the composition is both times a rotation by 180° (=point reflection), and indeed one order equals a rotation by 180°, the other by -180°, which is the same.

The composition of three or more reflections is associative, that means $S_i \bullet (S_h \bullet S_g) = (S_i \bullet S_h) \bullet S_g$.

(ii) Let g and h be **two parallel straight lines** with the (oriented) perpendicular **a from** g **to** h. Let k and l be two more parallel lines, also with the (oriented) perpendicular a from k to l (so k and l are parallel to g and h). Then $S_l \bullet S_k$ is equal to the translation $V_{2\cdot \mathbf{a}}$ as $S_h \bullet S_g$, so $S_l \bullet S_k = S_h \bullet S_g$.

Conversely, if you have a translation $V_{\mathbf{b}}$, you can substitute it by the product of two reflections $S_h \bullet S_g$. The two axes need to be chosen as follows: They have to be perpendicular to **b**, that means in particular parallel to each other; and **the length of the perpendicular from** g **to** h must be $\frac{b}{2}$. — If **these conditions** are met, then the axes can be **anywhere in the plane**; — both reflections, performed in the correct order, result in the given translation.

(iii) Let g and h be **two non-parallel straight lines** with the intersection Z and measure w of the (oriented) angle from **g to h** (-90°<w<90° , w≠0°). Let k and l be **two other non-parallel lines**, also with the intersection Z and the same measure w of the (oriented) angle from k to l. Then $S_l \bullet S_k$ is equal to the rotation $D_{Z;2\cdot w}$ as $S_h \bullet S_g$, $S_l \bullet S_k = S_h \bullet S_g$.

Conversely, if you have a rotation $D_{Y;u}$, you can substitute it by the product of two reflections $S_h \bullet S_g$. Besides the case u=0°, the two axes need to be chosen as follows: Both axes have to pass through Y, and the measure of the **angle from** g **to** h has to be $\frac{u}{2}$ (in particular, it needs to have the same algebraic sign as u). — If **these conditions** are met, then the axes can **lie in any direction** through Y; — both reflections, performed in the correct order, result in the given rotation.

…

(vi) A product $S_n \bullet S_{n1} \bullet \ldots \bullet S_1$ of n reflections S_1, S_2, ..., S_n (n>3) is equal to the product $T_m \bullet T_{m1} \bullet \ldots \bullet T_1$ of m (in generally other) reflections T_1, T_2, ..., T_m with m = n - 2. This decrement by 2 reflections can be continued as long as there are more than 3, **and finally every product of n reflections is equal to a product of at most three reflections** (for even n of at most **two**) reflections.

The statement in conclusion (vi) is a special case of the three-reflection theorem we already know; it is now proved again constructively for the case of 4 reflections

We justify this statement using a configuration of 4 reflections as an example. For the sake of clarity, we do not designate the lines of reflection with lower case letters this time, but number the reflections according to the formulation in the statement above. Therefore, in this case the axes of reflections are designated with the numbers 1-4. S_1 denotes the first reflection at axis with the number 1 which is performed in the composition of the four reflections.

Compared to the first image of Fig. I.3.14, S_1 and S_2 are replaced by $S_{1'}$ and $S_{2'}$ in the second image, preserving intersection point A and angular measure u, in a way that axis 2' passes through intersection point B of axes 3 and 4. In the third image, S_3 and S_4 are replaced by $S_{3'}$ and $S_{4'}$, preserving intersection point B and angular measure, in a way that axes 3' and 2' are identical.

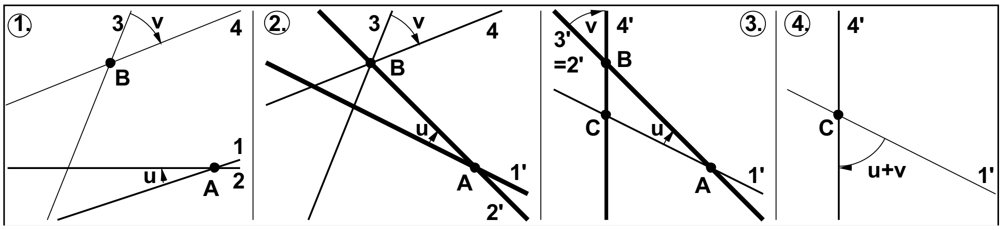

Abb. I.3.14: Product of 4 reflections is replaced by a product of 2 reflections.

Then

$$S_4 \bullet (S_3 \bullet (S_2 \bullet S_1)) = (S_4 \bullet S_3) \bullet (S_2 \bullet S_1) = (S_{4'} \bullet S_{3'}) \bullet (S_{2'} \bullet S_{1'}) =$$

$$S_{4'} \bullet ((S_{3'} \bullet S_{2'}) \bullet S_{1'}) = (S_{4'} \bullet \mathrm{I}) \bullet S_{1'} = S_{4'} \bullet S_{1'} ,$$

—and this is a rotation (fourth image) (in special cases a translation). While the measure of the rotation angle is relatively easy to identify, namely as sum u+v of the angle measures u and v of the two rotations $S_4 \bullet S_3$ and $S_2 \bullet S_1$, the determination of the center of rotation is more complicated. Here, one has initially the product of two rotations $D_{A;2\cdot u}$ and $D_{B;2\cdot v}$ and obtains as a result the rotation $D_{C;2\cdot(u+v)}$. — For our purposes, it is essential to have succeeded in writing a product of 4 reflections as an **equal** product of 2 reflections.

📎 Close-Reading-Task 08

…

📎 Homework Task & Problem 10

Appendix C

Overview of the RSV "Comprehending mathematical argumentation"

**Time (min:sec)**	**Operation**	**Strategy**	**Aim**	**Content**	**Quote from the video**	**Screenshot**
**0:09 – 0:24**	Presenting the learning intentions	Learning intentions (Fisher & Frey, 2016)	Making the goal of the RSV transparent to students	Goals of the RSV		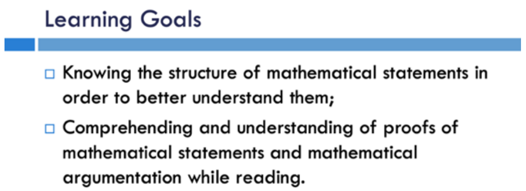
**0:25 – 1:52**	Presenting the structure of mathematical statement. Highlighting premises and their indicators in green and conclusions and their indicators in red, exemplified by if-then structures	Understand the theorem statement (Weber, 2015)	Providing knowledge about the structure of mathematical statements in order to better understand them	The product of two reflections in non-parallel lines but intersection point Z	"Mathematical statements are implications, that is, logical compositions, between two statements."	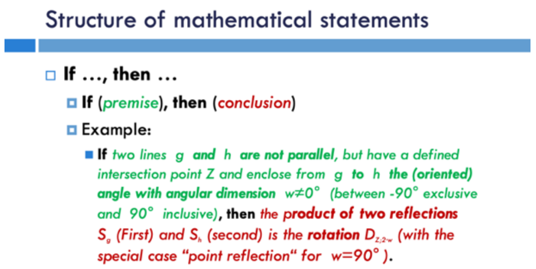
**1:52 – 3:15**	Highlighting premises and their indicators in green and conclusions and their indicators in red, exemplified by alternative phraseologies	Understand the theorem statement (Weber, 2015)	Providing knowledge about the structure of mathematical statements in order to better understand them	The product of two reflections in non-parallel lines but intersection point Z	"This if-then structure is not always expressed linguistically in this way. Alternative phraseologies are 'provided certain conditions hold, then a certain statement is or is valid.' Sometimes the order of the statements is also reversed. In the sentence about the	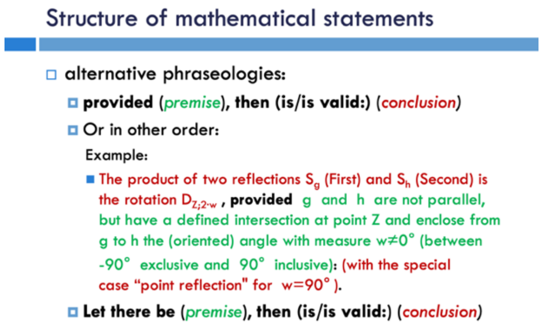

Time (min:sec)	Operation	Strategy	Aim	Content	Quote from the video	Screenshot
					product of two reflections as it is written in the script, this is exactly the case. Likewise, one finds phraseologies as ‘let there be’ certain conditions given, then a certain statement is or is valid.”	
**3:16 – 5:25**	Highlighting premises in green, conclusions in red, and associated indicators in yellow, exemplified by reading a script section sentence by sentence	Understand the theorem statement (Weber, 2015)	Applying provided knowledge about the structure of mathematical statements to the script in order to foster understanding	Conclusions of the theorem of the product of two reflections in parallel lines	“There [in the script] we find the phrase ‘let there be’ as a marker for a premise…. ‘Then’ follows the statement.”	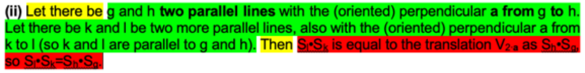 **(ii)** Let there be g and h **two parallel lines** with the (oriented) perpendicular **a from** g **to** h. Let there be k and l be two more parallel lines, also with the (oriented) perpendicular a from k to l (so k and l are parallel to g and h). Then $S_l \cdot S_k$ is equal to the translation $V_{2\cdot a}$ as $S_h \cdot S_g$, so $S_l \cdot S_k = S_h \cdot S_g$.
**5:26 – 7:09**	Exemplifying the mathematical theorem by developing a geometrical construction of the situation based on key statements of the theorem	Using examples to make sense of statements within the proof (Weber, 2015). Exploring the content using multiple representations (Shanahan et al., 2011)	Comprehending the mathematical statement by developing a geometrical model of the situation using examples	Conclusion of the theorem about the product of two reflections in parallel lines	“We need two parallel straight lines g and h with the oriented perpendicular a from g to h as well as two further parallel straight lines k and l also with the oriented perpendicular a from k to l.”	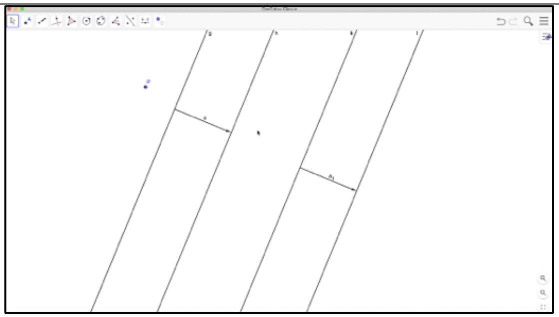
**7:10 – 8:25**	Rereading	Understand the theorem statement, put a strong emphasis on accuracy	Comprehending mathematical statements while reading	The product of two reflections in parallel lines f and g is a translation	“This statement is completely independent of the position of the two straight lines g and h in the plane.… The	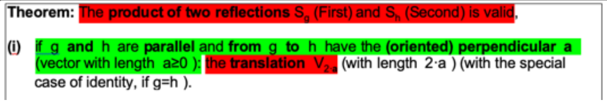 **Theorem:** The **product of two reflections** S_g (First) and S_h (Second) is valid, **(i)** if g **and** h are **parallel** and **from** g **to** h have the **(oriented) perpendicular a** (vector with length a≥0): the **translation** $V_{2\cdot a}$ (with length 2·a) (with the special case of identity, if g=h).

Time (min:sec)	Operation	Strategy	Aim	Content	Quote from the video	Screenshot
		(Shanahan et al., 2011)		with the double distance from f to g	theorem already contains the statement; it was only emphasized once more. Only by pausing we have recognized this more exactly.”	
**8:26 – 9:07**	Highlighting premises and their indicators in green and conclusions and their indicators in red	Understand the theorem statement (Weber, 2015)	Analysis of the structure of mathematical statements	Justification of the reduction strategy using the example of reflections in four straight lines	“Given a product of n reflections, that product can be replaced by another product of reflections, where the number of reflections is less by two.”	**(vi)** A product $S_n \cdot S_{n1} \cdot \ldots \cdot S_1$ of n reflections S_1, S_2, ..., S_n (n>3) is equal the product $T_m \cdot T_{m1} \cdot \ldots \cdot T_1$ of m (in generally other) reflections T_1, T_2, ..., T_m with m=n-2. This dec-rement by 2 reflections can be continued as long as there are more than 3, **and finally every product of n reflections is equal to a product of at most three reflections** (for even n of at most **two**) reflections.
**9:08 – 12:26**	Asking why with every illustration of the linking reflections and comparing it to a former mathematical theorem	Partitioning the proof into parts (Weber, 2015) Making connections (Berger, 2019a)	Justifying and comprehending mathematical statements while reading	Justification of the reduction strategy using the example of reflections in four straight lines	“To understand the rationale, we need to justify each step by asking ourselves ‘Why is this true?’… We find the answer above [in the script].”	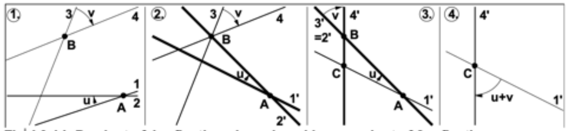 Fig. I.3.14: Product of 4 reflections is replaced by a product of 2 reflections.
**12:27 – 16:02**	Asking oneself why at every equal sign of the chain of equations and comparing it to former illustrations	Making connections (Berger, 2019a)	Justifying and comprehending mathematical statements while reading	Justification of the reduction strategy using the example of reflections in four straight lines	“What have we achieved now through this action? Let’s look at this in the individual steps of the chain of equations.… Overall, we have accounted for every single step through previous content of the script.”	$S_4 \cdot (S_3 \cdot (S_2 \cdot S_1)) = (S_4 \cdot S_3) \cdot (S_2 \cdot S_1) = (S_{4'} \cdot S_{3'}) \cdot (S_{2'} \cdot S_{1'}) = S_{4'} \cdot ((S_{3'} \cdot S_{2'}) \cdot S_{1'}) = (S_{4'} \cdot I) \cdot S_{1'} = S_{4'} \cdot S_{1'}$,
